# Treatment Outcomes From Erlotinib and Gefitinib in Advanced Epidermal Growth Factor Receptor–Mutated Nonsquamous Non–Small Cell Lung Cancer in Aotearoa New Zealand From 2010 to 2020: Nationwide Whole-of-Patient-Population Retrospective Cohort Study

**DOI:** 10.2196/65118

**Published:** 2025-03-03

**Authors:** Phyu Sin Aye, Joanne Barnes, George Laking, Laird Cameron, Malcolm Anderson, Brendan Luey, Stephen Delany, Dean Harris, Blair McLaren, Elliott Brenman, Jayden Wong, Ross Lawrenson, Michael Arendse, Sandar Tin Tin, Mark Elwood, Philip Hope, Mark James McKeage

**Affiliations:** 1 Department of Pharmacology and Clinical Pharmacology, University of Auckland, Auckland, New Zealand; 2 School of Pharmacy, University of Auckland, Auckland, New Zealand; 3 Te Aka Mātauranga Matepukupuku Centre for Cancer Research, University of Auckland, Auckland, New Zealand; 4 Department of Medical Oncology, Te Pūriri o Te Ora Regional Cancer and Blood Service, Te Whatu Ora Health New Zealand, Auckland City Hospital, Auckland, New Zealand; 5 Department of Medical Oncology, Te Whatu Ora Health New Zealand Te Pae Hauuora o Ruahine o Tararua, Palmerston North Hospital, Palmerston North, New Zealand; 6 Wellington Blood and Cancer Centre, Te Whatu Ora Health New Zealand Capital, Coast and Hutt Valley, Wellington Hospital, Wellington, New Zealand; 7 Department of Oncology, Te Whatu Ora Health New Zealand Nelson Marlborough, Nelson Hospital, Nelson, New Zealand; 8 Oncology Service, Te Whatu Ora—Waitaha Canterbury, Christchurch Hospital, Christchurch, New Zealand; 9 Southern Blood and Cancer Service, Te Whatu Ora Southern, Dunedin Hospital, Dunedin, New Zealand; 10 Cancer and Haematology Services, Te Whatu Ora Health New Zealand Haora a Toi Bay of Plenty, Tauranga Hospital, Tauranga, New Zealand; 11 Cancer Services, Te Whatu Ora Health New Zealand Waikato, Waikato Hospital, Hamilton, New Zealand; 12 Medical Research Centre, University of Waikato, Hamilton, New Zealand; 13 Department of Pathology, Te Whatu Ora Health New Zealand Waikato, Waikato Hospital, Hamilton, New Zealand; 14 Department of Epidemiology and Biostatistics, University of Auckland, Auckland, New Zealand; 15 Lung Foundation New Zealand, Auckland, New Zealand

**Keywords:** non–small cell lung cancer, mutations, epidemiology, target therapy, retrospective cohort study

## Abstract

**Background:**

Health care system–wide outcomes from routine treatment with erlotinib and gefitinib are incompletely understood.

**Objective:**

The aim of the study is to describe the effectiveness of erlotinib and gefitinib during the first decade of their routine use for treating advanced epidermal growth factor receptor (*EGFR*) mutation-positive nonsquamous non–small cell lung cancer in the entire cohort of patients treated in Aotearoa New Zealand.

**Methods:**

Patients were identified, and data collated from national pharmaceutical dispensing, cancer registration, and mortality registration electronic databases by deterministic data linkage using National Health Index numbers. Time-to-treatment discontinuation and overall survival were measured from the date of first dispensing of erlotinib or gefitinib and analyzed by Kaplan-Meier curves. Associations of treatment outcomes with baseline factors were evaluated using univariable and multivariable Cox regressions.

**Results:**

Overall, 752 patients were included who started treatment with erlotinib (n=418) or gefitinib (n=334) before October 2020. Median time-to-treatment discontinuation was 11.6 (95% CI 10.8‐12.4) months, and median overall survival was 20.1 (95% CI 18.1‐21.6) months. Shorter time-to-treatment discontinuation was independently associated with high socioeconomic deprivation (hazard ratio [HR] 1.3, 95% CI 1.1‐1.5 compared to the New Zealand Index of Deprivation 1‐4 group), *EGFR* L858R mutations (HR 1.3, 95% CI 1.1‐1.6 compared to exon 19 deletion), and distant disease at cancer diagnosis (HR 1.4, 95% CI 1.2‐1.7 compared to localized or regional disease). The same factors were independently associated with shorter overall survival. Outcome estimates and predictors remained unchanged in sensitivity analyses.

**Conclusions:**

Outcomes from routine treatment with erlotinib and gefitinib in New Zealand patients with advanced *EGFR*-mutant nonsquamous non–small cell lung cancer are comparable with those reported in randomized trials and other health care system–wide retrospective cohort studies. Socioeconomic status, *EGFR* mutation subtype, and disease extent at cancer diagnosis were independent predictors of treatment outcomes in that setting.

## Introduction

Lung cancer is the most common cause of cancer death in the world today [1]. Most clinical presentations of lung cancer are nonsquamous non–small cell lung cancer (NSCLC) [2]. Epidermal growth factor receptor (*EGFR*)–mutant nonsquamous NSCLC was the first type of lung cancer identified with an oncogenic driver that could be directly targeted by drug treatment [3-5].

The treatment of advanced *EGFR*-mutant nonsquamous NSCLC has evolved rapidly following the results of randomized controlled trials demonstrating improved progression-free survival. Initial randomized controlled trials established the superiority of first-generation *EGFR* kinase inhibitors, erlotinib and gefitinib, over platinum-doublet chemotherapy [6-10]. Subsequent randomized controlled trials established the superiority of second- and third-generation *EGFR* kinase inhibitors, including afatinib, dacomitinib, osimertinib, aumolertinib, and lazertinib, over those first-generation inhibitors [11-15]. Other randomized controlled trials compared erlotinib or gefitinib given alone or in combination with bevacizumab, ramucirumab, or chemotherapy [16-20]. In the 15 aforementioned randomized controlled trials, a total of 2257 patients with advanced *EGFR*-mutant nonsquamous NSCLC were allocated erlotinib or gefitinib monotherapy in control or experimental treatment arms. In those erlotinib or gefitinib monotherapy treatment arms, median progression-free survival ranged from 8.0 to 13.3 months. These clinical trial data provide a point of reference against which real-world studies of outcomes from treatment with erlotinib or gefitinib can be compared.

To fully understand outcomes from treatment with erlotinib and gefitinib in the setting of routine care, large-scale observational studies are required in addition to the extensive data already available from randomized controlled trials. Randomized controlled trials may have overestimated the benefits [21], and underestimated the harms [22], associated with the routine use of erlotinib and gefitinib. Compared to participants in randomized controlled trials, patients presenting for routine treatment with erlotinib or gefitinib are older, are of non-Asian ethnicity, have more comorbidities, have poorer performance status, and more often have brain metastasis. Randomized controlled trials have not evaluated many factors potentially impacting treatment outcomes, such as socioeconomic status. To improve their generalizability and avoid bias, observational studies of real-world outcomes from treatment with erlotinib and gefitinib could include all patients treated within a whole health care system or nation rather than being limited to those from 1 or a few institutions. To aid comparisons to clinical trial data, those observational studies could evaluate progression-free survival or proxies of progression-free survival rather than just overall survival, which is strongly influenced by factors other than treatment with erlotinib or gefitinib.

Only since 2019 have large-scale nationwide or health care system–wide studies reported real-world outcomes from routine treatment with erlotinib and gefitinib in patients with advanced *EGFR*-mutant NSCLC from Canada [23], the United States [24], Taiwan [25,26], Poland [27], Finland [28], and the Netherlands [29,30]. Among those aforementioned studies, 5 studies [24-28] reported progression-free survival or proxies of progression-free survival, such as time-to-treatment failure. In those 5 studies, median progression-free survival or its proxy ranged from 9.7 to 13.1 months. These observational data provide a point of reference against which other real-world studies of outcomes from treatment with erlotinib or gefitinib can be compared.

Starting in 2010, erlotinib and gefitinib were introduced into routine use in Aotearoa New Zealand for treating advanced lung cancer. The overall *EGFR* mutation positivity among patients with nonsquamous NSCLC who were tested was 22.5% in New Zealand [31]. To date, the effectiveness of erlotinib and gefitinib in the general population of New Zealand patients with lung cancer has not been described. With this background, this study aimed to describe the effectiveness of erlotinib and gefitinib during the first decade of their routine use for the treatment of advanced *EGFR*-mutant nonsquamous NSCLC in the entire cohort of patients treated in New Zealand. The study also aimed to evaluate associations between baseline factors and the effectiveness of erlotinib and gefitinib in this real-world setting.

## Methods

### Study Design and Participants

This was a nationwide, population-based, observational, data-linkage, retrospective cohort study that analyzed routinely collected health and administrative electronic data. The study group was a whole-of-population sample comprising a single group of patients. Patients were eligible for inclusion if they (1) were diagnosed with *EGFR*-mutant lung cancer, (2) dispensed erlotinib or gefitinib first before October 1, 2020, and (3) followed thereafter until death or for at least 1 year. Patients were excluded from the study if they had (1) erlotinib dispensed before January 1, 2014, or gefitinib dispensed before August 1, 2012, when positive *EGFR* mutation test results became mandatory for state-subsidized treatment; (2) no notification of a diagnosis of nonsquamous NSCLC in the New Zealand Cancer Registry; or (3) an unactionable or unknown *EGFR* mutation subtype.

### Setting

From 2010 to 2020, New Zealand had a resident population ranging from approximately 4.3 to 5.1 million people, comprising predominately New Zealand European (70%), Māori (17%), Asian (15%), and Pacific people (8%) [32] (the total percent is greater than 100 because some people have more than 1 self-reported ethnicity). New Zealand residents were eligible for state-funded health care, including state-subsidized prescription medicines. Starting in 2010, the *EGFR* kinase inhibitor drugs erlotinib and gefitinib were introduced into routine clinical use in New Zealand for lung cancer treatment [33]. From October 1, 2010, to December 31, 2013, erlotinib was state-funded as a second-line treatment for advanced NSCLC, initially without any requirement for *EGFR* mutation testing. From August 1, 2012, gefitinib was state-funded as a first-line treatment for advanced *EGFR*-mutant NSCLC in New Zealand. On May 1, 2013, the National Health Committee of the New Zealand Ministry of Health issued recommendations for *EGFR* mutation testing in New Zealand, including testing of all patients with nonsquamous NSCLC at diagnosis irrespective of stage as part of standard pathology processes. From January 1, 2014, state funding for erlotinib was restricted to treating advanced *EGFR*-mutant NSCLC in New Zealand. During the first decade of routine use of erlotinib and gefitinib for lung cancer treatment in New Zealand, from 2010 to 2020, no other *EGFR* kinase inhibitor drugs were state-funded for use in New Zealand. During the period of study, treatment with erlotinib or gefitinib was provided by 10 public hospitals, and *EGFR* mutation testing was provided by 3 pathology laboratories in New Zealand.

### Ethical Considerations

Ethics approval for this study was obtained from the New Zealand Government Ministry of Health Northern B Health and Disability Ethics Committee (reference 13/NTB/165/AM02). As the research retrospectively analyzed routinely collected data and did not involve direct contact with patients, the participants were not able or required to give informed consent by the ethics committee or governance groups who approved the study. The study used the identifiable data, which were password-protected, stored on the secured University of Auckland managed drive, and only accessible to the research team. The study was registered (ACTRN12615000998549). A study protocol and results of a validation substudy have been published [34].

### Data Sources

Patients were identified, and data collated from national electronic pharmaceutical dispensing (Pharmaceutical Information Database [PHARMs]), cancer registration (New Zealand Cancer Registry), and mortality registration (National Mortality Collection) databases. Individual-level data on eligible cohort patients were compiled from these national electronic health databases by deterministic data linkage using each patient’s unique National Health Index number. Additional data on eligible cohort patients were sourced from regional laboratory test data repositories, databases, and clinical records to determine the *EGFR* mutation status. A validation substudy demonstrated the feasibility and validity of using these national electronic health databases as the main source of data for this study [34].

### Outcomes

The primary effectiveness outcome for this analysis was time-to-treatment discontinuation. Prescribing guidelines [35,36] recommend continuing daily treatment with erlotinib or gefitinib until disease progression, as long as treatment is safe and tolerable. In an analysis of randomized clinical trials submitted to the Food and Drug Administration, time-to-treatment discontinuation correlated well (*r*=0.91) with progression-free survival for patients with advanced *EGFR*-mutant NSCLC treated with *EGFR* kinase inhibitor drugs [37]. Time-to-treatment discontinuation is also less affected by subsequent cancer treatments and other factors that impact overall survival. Time-to-treatment discontinuation thereby reflects the duration of benefit from treatment with erlotinib or gefitinib. Time-to-treatment discontinuation was defined as the duration between the dates of the first dispensing and the last treatment with erlotinib or gefitinib. The date of last treatment with erlotinib or gefitinib was calculated by adding the number of days erlotinib or gefitinib dispensed for at the last dispensing to the date of the last dispensing, except when death occurred before the calculated date of last treatment, in which case the date of last treatment was the date of death. The secondary effectiveness outcome for this analysis was overall survival, defined as the duration between the date of first dispensing of erlotinib or gefitinib and death from any cause. A validation substudy had demonstrated the feasibility and validity of these methodologies for determining the outcomes of this study [34].

### Variables

Baseline variables used for patient characterization included age, sex, ethnicity, geographical region of residence, smoking status, performance status, diagnosis year, NSCLC morphology, basis of NSCLC diagnosis, disease extent at cancer diagnosis, socioeconomic deprivation, rurality, comorbidity, choice of erlotinib or gefitinib for initial treatment, and *EGFR* mutation subtype. Socioeconomic deprivation was determined by mapping domicile codes recorded in the New Zealand Cancer Registry to the 2006 New Zealand Index of Deprivation and was categorized into deciles with 1 being the least deprived and 10 being the most deprived [38]. Rurality was determined using the same domicile codes applied to Statistics New Zealand’s Urban/Rural profile [39]. Comorbidity was assessed using a validated pharmacy-based comorbidity index for patients with cancer [40], modified for this study as previously described [34]. Ethnicity was classified into Asian, Māori, New Zealand European, or Pacific, and prioritized ethnicity was used if a registration listed multiple ethnicities (patients with more than 1 recorded ethnicity were allocated to a single ethnic group in order of priority: Māori, Pacific, Asian, and New Zealand European) [41]. *EGFR* mutation variants were classified according to the system of Koopman et al [42] into the following categories: (1) exon 19 deletion, (2) L858R, (3) uncommon actionable variant, (4) exon 20 insertion, and (5) nonactionable or unknown variant. Since *EGFR* mutation variant categories (4) and (5) were unactionable with erlotinib or gefitinib, patients with those variants were excluded from this study. A validation substudy had demonstrated the feasibility and validity of the methodologies used for determining the variables used for this study [34].

### Literature Search

For comparing the results from this study to those from randomized controlled trials and other retrospective observational studies, a literature search was undertaken using a combination of the following MeSH terms: “carcinoma, non-small-cell lung,” “ErbB receptors,” “erlotinib hydrochloride,” “gefitinib,” “protein kinase inhibitors,” and “mutation.” Observational studies were included in this comparison if they were nationwide or health care system–wide studies and reported progression-free survival, or a proxy of progression-free survival, measured from the commencement of treatment with erlotinib or gefitinib [24-28]. Institution-based studies [43] and those not reporting progression-free survival or a proxy of progression-free survival [23,29,30] were excluded from these comparisons.

### Statistical Analysis

Descriptive statistics were used to analyze the demographic profile and baseline characteristics of the retrospective cohort. Time-to-treatment discontinuation and overall survival were analyzed by Kaplan-Meier curves, and survival differences between subgroups were assessed using log-rank tests. Patients with no known dates of last treatment or death were censored at the date of last follow-up of dispensing (June 30, 2022) or survival (May 7, 2022), respectively. To assess the robustness of estimates of time-to-treatment discontinuation and overall survival, sensitivity analyses were carried out in an expanded study cohort (n=885) that included patients with no registration of nonsquamous NSCLC and those with unknown or nonactionable *EGFR* mutation subtypes, except those with exon 20 insertions. Associations between baseline factors and time-to-treatment discontinuation or overall survival were evaluated by univariable and multivariable Cox regression models to compute hazard ratios and their 95% CIs and *P* values. Baseline factors selected for univariable and multivariable analyses included age, sex, disease morphology, disease extent, *EGFR* mutation subtype, and initial choice of *EGFR* kinase inhibitor drug, which had been identified as independent predictors of outcomes in previous studies [44,45], and ethnicity, comorbidity, socioeconomic deprivation, and residential status (urban vs or rural, and region), which had not been previously evaluated in the New Zealand patient population. There were complete data for all those factors for all 752 cohort patients. Smoking and Eastern Cooperative Oncology Group performance status were excluded from the univariable and multivariable analyses due to high levels of missing data (>50%). Missing extent of disease at cancer diagnosis and ethnicity data were included in univariable and multivariable analyses by adding an unknown category for each of these variables comprising <20% and <1% of patients, respectively. Otherwise, data were complete for all other factors for all 752 patients. Factors were selected for multivariable analyses if they had statistically significant associations with the outcome of interest on univariable analysis. To assess the robustness of the findings of multivariable analyses, sensitivity analyses were carried out using less stringent criteria for factor inclusion. Differences were considered statistically significant when *P* values were less than .05. Data analyses were performed using Stata (version 16; StataCorp LLC).

## Results

**Figure 1.**
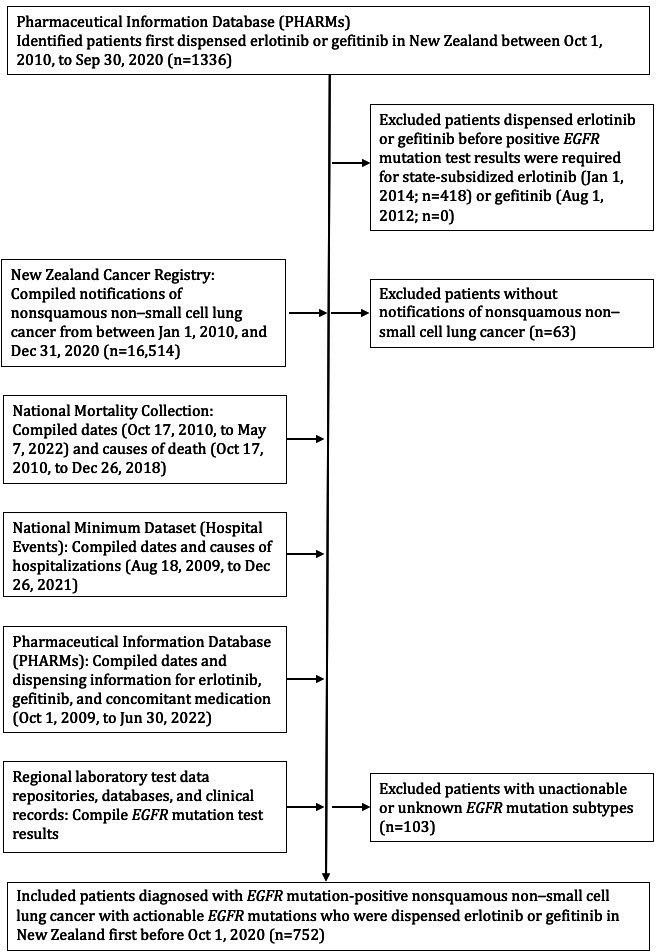
CONSORT (Consolidated Standards of Reporting Trials) flow diagram of the assembly of the cohort and data collation. *EGFR*: epidermal growth factor receptor.

The assembly of the retrospective cohort and compilation of study data from national electronic health databases was carried out as shown in Figure 1. From the PHARMs, 1336 patients were identified who had been dispensed erlotinib or gefitinib first between October 1, 2010, and September 30, 2020. A total of 418 of those patients were excluded because they were first dispensed erlotinib or gefitinib before positive *EGFR* mutation test results became mandatory for access to state-subsidized erlotinib or gefitinib in New Zealand, leaving 918 potentially eligible patients. From the New Zealand Cancer Registry, 16,516 patients were identified with notifications of nonsquamous NSCLC diagnoses made between January 1, 2010, and December 30, 2020. Of the 918 potentially eligible patients, 63 did not have notifications of diagnoses of nonsquamous NSCLC recorded in the New Zealand Cancer Registry and were excluded, leaving 855 potentially eligible patients. Dates and causes of death and hospitalizations and full dispensing information for erlotinib, gefitinib, and concomitant medications were compiled on those patients from the National Mortality Collection, National Minimum Dataset (Hospital Events), and PHARMs, respectively. *EGFR* mutation test results, smoking status, and Eastern Cooperative Oncology Group performance status were then compiled from regional laboratory test data repositories, databases, and clinical records. Of 855 potentially eligible patients, 103 patients had unactionable or unknown *EGFR* mutation variants, including 33 patients with *EGFR* exon 20 insertions, and were excluded. Finally, 752 patients remained, who had been diagnosed with *EGFR*-mutant nonsquamous NSCLC with actionable *EGFR* mutation variants, and had started treatment with erlotinib or gefitinib prior to October 2020 for inclusion in this study.

The baseline characteristics of the retrospective 752 patient cohorts are shown in Table 1. Their mean age was 67 (SD 12) years, and 67% (n=504) were female. About one-quarter were Asian, half New Zealand European, one-quarter Pacific or Māori, and 1.1% (n=8) had other or unknown ethnicity. Most had adenocarcinoma and *EGFR* exon 19 deletions or L858R mutations. The extent of disease at cancer diagnosis was available for only 604 (80.3%) patients, most of whom had distant disease at cancer diagnosis. Smoking status was available for only 301 (40.1%) patients, most of whom were nonsmokers. Performance status was available for only 273 (36.6%) patients, most of whom had limited performance status.

**Table 1.** Patient characteristics (N=752).

		Values, n (%)
**Age (years)^a^**		
	<65	318 (42.3)
	65+	434 (57.7)
**Sex**		
	Male	248 (33)
	Female	504 (67)
**Ethnicity**		
	Asian	190 (25.3)
	Māori	73 (9.7)
	New Zealand European	392 (52.1)
	Pacific	89 (11.8)
	Other and unknown	8 (1.1)
**Region**		
	Northern	355 (47.2)
	Midland	103 (13.7)
	Others	294 (39.1)
**Smoking**		
	Ex-smoker	108 (14.4)
	Nonsmoker	160 (21.3)
	Current smoker	33 (4.4)
	Unknown	451 (59.9)
**ECOG^b^ performance status**		
	Fully active (0)	124 (16.5)
	Limited (1-4)	151 (20.1)
	Unknown	477 (63.4)
**Diagnosis year**		
	2010‐2013	120 (16)
	2014‐2016	285 (37.9)
	2017‐2020	347 (46.1)
**Morphology**		
	Adenocarcinoma	662 (88)
	Unspecified and other	90 (12)
**Basis of diagnosis**		
	Histology	459 (61)
	Cytology	275 (36.6)
	Other	18 (2.4)
**Extent**		
	Localized or regional	136 (18.1)
	Distant	468 (62.2)
	Unknown	148 (19.7)
**Deprivation**		
	NZDep^c^ 1‐4	305 (40.6)
	NZDep 5‐7	218 (29)
	NZDep 8‐10	229 (30.5)
**Rurality**		
	Urban	652 (86.7)
	Rural	100 (13.3)
**Comorbidity**		
	No	190 (25.3)
	Yes	562 (74.7)
***EGFR*^d^ type**		
	Exon 19 del	424 (56.4)
	Exon 21 L858R	256 (34)
	Uncommon or actionable	72 (9.6)
***EGFR*-TKI^e^**		
	Gefitinib	334 (44.4)
	Erlotinib	418 (55.6)

a Mean age 67 (SD 12, range 24‐92) years.

b ECOG: Eastern Cooperative Oncology Group.

c NZDep: New Zealand Index of Deprivation.

d *EGFR*: epidermal growth factor receptor.

e TKI: tyrosine kinase inhibitor.

At the date of the last dispensing follow-up (June 30, 2022), treatment with erlotinib and gefitinib had been discontinued in 724 (96.3%) patients and was continuing in 28 (3.7%) patients. Treatment was discontinued prior to death in 618 patients and at the time of death in 103 patients. Median time-to-treatment discontinuation was 11.6 (95% CI 10.8-12.4) months. The 1-, 2-, and 5-year rates of treatment continuation were 47.3% (95% CI 34.7%-50.9%), 17.4% (95% CI 14%-20.2%), and 3.4% (95% CI 2.2%-5.1%), respectively. Sensitivity analysis in an expanded study cohort (n=885) gave similar results for median time-to-treatment discontinuation (11.1, 95% CI 10.1-11.8 months). Univariable analysis (Table 2) showed that shorter time-to-treatment discontinuation was associated with socioeconomic deprivation, *EGFR* L858R mutations, distant disease at cancer diagnosis, and adenocarcinoma morphology. The initial choice of *EGFR* kinase inhibitor (erlotinib or gefitinib), age, sex, ethnicity, geographical region, year of diagnosis, basis of diagnosis, rurality, and comorbidity were not associated with time-to-treatment discontinuation on univariable analysis. Multivariable analysis showed that shorter time-to-treatment discontinuation was independently associated with socioeconomic deprivation, *EGFR* L858R mutations, and distant disease at cancer diagnosis (Table 2). Sensitivity analyses using less stringent criteria for factor inclusion identified the same independent predictors of time-to-treatment discontinuation (Table S3 in Multimedia Appendix 1).

**Table 2.** Univariable and multivariable analysis of time-to-treatment discontinuation.

		Univariable analysis		Multivariable analysis	
		HR^a^ (95% CI)	*P* value	HR (95% CI)	*P* value
***EGFR*^b^-TKI^c^**					
	Gefitinib	1.1 (1.0‐1.3)	.07	—^d^	—
	Erlotinib	1.0 (—)	—	—	—
**Age (years)**					
	<65	1.0 (0.9‐1.2)	.63	—	—
	65+	1.0 (—)	—	—	—
**Sex**					
	Male	1.0 (0.8‐1.1)	.74	—	—
	Female	1.0 (—)	—	—	—
**Ethnicity**					
	Asian	0.9 (0.7‐1.0)	.14	—	—
	Māori	1.1 (0.9‐1.4)	.37	—	—
	New Zealand European	1.0 (—)	—	—	—
	Pacific	1.0 (0.8‐1.2)	.76	—	—
	Other and unknown	0.8 (0.4‐1.7)	.58	—	—
**Region**					
	Northern	1.0 (—)	—	—	—
	Midland	1.1 (0.9‐1.4)	.45	—	—
	Others	1.0 (0.9‐1.2)	.95	—	—
**Diagnosis year**					
	2010‐2013	1.0 (0.8‐1.2)	.92	—	—
	2014‐2016	0.9 (0.8‐1.1)	.17	—	—
	2017‐2020	1.0 (—)	—	—	—
**Morphology**					
	Adenocarcinoma	1.0 (—)	—	1.0 (—)	—
	Unspecified and other	0.8 (0.6‐1.0)	.04	0.9 (0.7‐1.1)	.25
**Basis of diagnosis**					
	Histology	1.0 (—)	—	—	—
	Cytology	1.1 (0.9‐1.3)	.29	—	—
	Other	0.9 (0.5‐1.4)	.58	—	—
**Extent**					
	Localized or regional	1.0 (—)	—	1.0 (—)	—
	Distant	1.5 (1.2‐1.8)	<.001	1.4 (1.2‐1.7)	.001
	Unknown	0.9 (0.7‐1.1)	.38	0.9 (0.7‐1.1)	.33
**Deprivation**					
	NZDep^e^ 1‐4	1.0 (—)	—	1.0 (—)	—
	NZDep 5‐7	1.2 (1.0‐1.5)	.02	1.2 (1.0‐1.4)	.06
	NZDep 8‐10	1.3 (1.1‐1.6)	.004	1.3 (1.1‐1.5)	.005
**Rurality**					
	Urban	1.0 (—)	—	—	—
	Rural	1.1 (0.9‐1.4)	.39	—	—
**Comorbidity**					
	No	1.2 (1.0‐1.4)	.11	—	—
	Yes	1.0 (—)	—	—	—
***EGFR* type**					
	Exon 19 deletion	1.0 (—)	—	1.0 (—)	—
	Exon 21 L858R	1.3 (1.1‐1.5)	.001	1.3 (1.1‐1.6)	<.001
	Uncommon or actionable	1.2 (0.9‐1.6)	.17	1.3 (1.0‐1.6)	.07

a HR: hazard ratio.

b *EGFR*: epidermal growth factor receptor.

c TKI: tyrosine kinase inhibitor.

d Not applicable.

e NZDep: New Zealand Index of Deprivation.

At the date of last survival follow-up (May 7, 2022), 614 (81.6%) patients had died, and 138 (18.4%) patients were alive. Median overall survival was 20.1 (95% CI 18.1-21.6) months. The 1-, 2-, and 5-year overall survival rates were 69.2% (95% CI 65.8%-72.4%), 43% (95% CI 37.4%-43.5%), and 13.9% (95% CI 11.2%-17%), respectively. Sensitivity analysis in an expanded study cohort (n=885) gave similar results for median overall survival (19.4, 95% CI 17.8-21.2 months). Univariable analysis (Table 3) showed shorter overall survival in association with socioeconomic deprivation, *EGFR* L858R mutations, distant disease at cancer diagnosis, initial choice of *EGFR* kinase inhibitor of gefitinib (vsversus erlotinib), age >65 years, non-Asian ethnicity, residence outside the Northern or Midlands regions, and adenocarcinoma morphology. Sex, diagnosis year, basis of diagnosis, rurality, and comorbidity were not associated with overall survival on univariable analysis. Multivariable analysis showed that shorter overall survival was independently associated with socioeconomic deprivation, *EGFR* L858R mutations, distant disease at cancer diagnosis, and non-Asian or non-Pacific ethnicities (Table 3). Sensitivity analyses using less stringent criteria for factor inclusion identified the same independent predictors of overall survival (Table S4 in Multimedia Appendix 1).

**Table 3.** Univariable and multivariable analysis of overall survival.

		Univariable analysis		Multivariable analysis	
		HR^a^ (95% CI)	*P* value	HR (95% CI)	*P* value
***EGFR*^b^-TKI^c^**					
	Gefitinib	1.2 (1.0‐1.4)	.02	1.2 (1.0‐1.4)	.10
	Erlotinib	1.0 (—^d^)	—	1.0 (—)	—
**Age (years)**					
	<65	0.9 (0.7‐1.0)	.048	0.9 (0.7‐1.0)	.14
	65+	1.0 (—)	—	1.0 (—)	—
**Sex**					
	Male	1.1 (0.9‐1.3)	.48	—	—
	Female	1.0 (—)	—	—	—
**Ethnicity**					
	Asian	0.7 (0.5‐0.8)	<.001	0.7 (0.6‐0.9)	<.001
	Māori	1.2 (0.9‐1.5)	.26	1.3 (1.0‐1.7)	.05
	New Zealand European	1.0 (—)	—	1.0 (—)	—
	Pacific	0.8 (0.6‐1.0)	.06	0.8 (0.6‐1.0)	.046
	Other and unknown	0.5 (0.2‐1.3)	.15	0.5 (0.2‐1.3)	.16
**Region**					
	Northern	1.0 (—)	—	1.0 (—)	—
	Midland	1.2 (0.9‐1.5)	.18	1.1 (0.8‐1.4)	.57
	Others	1.4 (1.1‐1.6)	<.001	1.2 (1.0‐1.5)	.06
**Diagnosis year**					
	2010‐2013	1.2 (0.9‐1.4)	.24	—	—
	2014‐2016	1.0 (0.8‐1.2)	.82	—	—
	2017‐2020	1.0 (—)	—	—	—
**Morphology**					
	Adenocarcinoma	1.0 (—)	—	1.0 (—)	—
	Unspecified and other	0.7 (0.6‐1.0)	.02	0.9 (0.7‐1.2)	.4
**Basis of diagnosis**					
	Histology	1.0 (—)	—	—	—
	Cytology	1.1 (0.9‐1.3)	.41	—	—
	Other	1.4 (0.9‐2.3)	.16	—	—
**Extent**					
	Localized or regional	1.0 (—)	—	1.0 (—)	—
	Distant	1.7 (1.4‐2.2)	<.001	1.8 (1.4‐2.2)	<.001
	Unknown	1.0 (0.8‐1.3)	.98	1.0 (0.7‐1.3)	.82
**Deprivation**					
	NZDep^e^ 1‐4	1.0 (—)	—	1.0 (—)	—
	NZDep 5‐7	1.4 (1.2‐1.7)	.001	1.3 (1.1‐1.6)	.006
	NZDep 8‐10	1.4 (1.1‐1.7)	.001	1.4 (1.1‐1.7)	.004
**Rurality**					
	Urban	1.0 (—)	—	—	—
	Rural	1.2 (1.0‐1.5)	.11	—	—
**Comorbidity**					
	No	1.0 (0.9‐1.3)	.67	—	—
	Yes	1.0 (—)	—	—	—
***EGFR* type**					
	Exon 19 deletion	1.0 (—)	—	1.0	—
	Exon 21 L858R	1.4 (1.2‐1.6)	<.001	1.5 (1.2‐1.7)	<.001
	Uncommon or actionable	1.3 (1.0‐1.7)	.09	1.2 (0.9‐1.6)	.18

a HR: hazard ratio.

b *EGFR*: epidermal growth factor receptor.

c TKI: tyrosine kinase inhibitor.

d Not applicable.

e NZDep: New Zealand Index of Deprivation.

For the purpose of comparison of the results from this study to the existing literature, our literature search identified 15 randomized controlled trials and 5 nationwide or health care system–wide retrospective observational studies. These randomized controlled trials showed that the median progression-free survival for erlotinib and gefitinib monotherapy treatment arms ranged from 8.0 to 13.3 months (Table S1 in Multimedia Appendix 1). The retrospective observational studies showed that the median progression-free survival, or its proxy, ranged from 9.7 to 13.1 months, and the median overall survival ranged from 17.5 to 23.9 months (Table S2 in Multimedia Appendix 1). Our study reports the results following the RECORD (Reporting of Studies Conducted Using Observational Routinely-Collected Health Data) statement checklist (Table S5 in Multimedia Appendix 1).

## Discussion

### Principal Findings and Comparison to Prior Work

The outcomes from treatment with erlotinib and gefitinib in this study of 752 patients with advanced *EGFR*-mutant nonsquamous NSCLC, treated between 2010 and 2020 in New Zealand, corresponded with those reported in randomized controlled trials and in other large-scale health care system–wide retrospective cohort analyses. The median time-to-treatment discontinuation of 11.6 months found in this study paralleled the median progression-free survival values reported for erlotinib and gefitinib monotherapy treatment arms of 15 randomized controlled trials [6-20], which ranged from 8.0 to 13.3 months (Table S1 in Multimedia Appendix 1). It also paralleled the median progression-free survival values, or its proxy, reported in other nationwide or health care system–wide observational studies of similar patient groups from elsewhere [24-28] (range of median progression-free survival or proxy 9.7 to 13.1 months; Table S2 in Multimedia Appendix 1). The median overall survival of 20.1 months found in this study was also within the range reported in other nationwide or health care system–wide observational studies of similar patient groups [24-28] (range of median overall survival 17.5 to 23.9 months; Table S2 in Multimedia Appendix 1). In this way, this retrospective study has confirmed that the therapeutic benefits expected from erlotinib and gefitinib had been conveyed into the setting of routine care in New Zealand.

*EGFR* mutation subtype was an independent predictor of outcomes from treatment with erlotinib and gefitinib in this study. Study patients were stratified according to whether their tumors had exon 19 deletions (56%), L858R mutations (34%), or other actionable *EGFR* mutations (10%). Compared to those with exon 19 deletions, study patients with L858R mutations had 30% and 50% increased risks of treatment discontinuation and death, respectively, after commencing treatment with erlotinib or gefitinib. This finding is consistent with those of previous studies exploring outcomes from erlotinib or gefitinib in similar patient groups [45]. *EGFR* mutation subtype may have impacted upon treatment outcomes in this study via the higher pharmacological potency of erlotinib and gefitinib for inhibiting exon 19 deletion *EGFR* oncoproteins compared to those associated with L858R or other *EGFR* mutations [46,47].

Socioeconomic deprivation was an independent predictor of outcomes from treatment with erlotinib and gefitinib in this study. Study patients were stratified into groups with low (41%), intermediate (29%), or high socioeconomic deprivation (30%) based on their residential area. Compared to the study patients from high socioeconomic areas, those from low socioeconomic areas had 30% and 50%, and those from intermediate socioeconomic areas had 20% and 30%, increased risks of treatment discontinuation and death, respectively, after commencing treatment with erlotinib or gefitinib. People from low socioeconomic areas are known to have poorer outcomes from lung cancer due to more limited access to screening and diagnostic services that lead to delayed diagnoses and more advanced disease at presentation [48]. However, few previous studies have evaluated the impacts of socioeconomic deprivation on outcomes from treatment with erlotinib, gefitinib, or other systemic anticancer therapies in patients with *EGFR*-mutant or other forms of advanced lung cancer. A pooled analysis of SWOG Cancer Research Network clinical trials showed significant associations between socioeconomic deprivation and lower progression-free survival, including in a subgroup of 1307 patients with stage IV NSCLC treated with various platinum-based chemotherapy regimens in randomized clinical trials [49]. Socioeconomic deprivation may have impacted treatment outcomes in this study by limiting access to health care during treatment with erlotinib and gefitinib, directly via other yet to be defined mechanisms or indirectly through correlated predictive factors not accounted for in the multivariable analyses, such as smoking and performance status. Future studies should more closely evaluate the impacts of socioeconomic deprivation on outcomes from the treatment of advanced lung cancer.

Disease extent at cancer diagnosis was an independent predictor of outcomes of treatment with erlotinib and gefitinib in this study. Study patients were stratified according to whether they had localized or regional (18%), distant (62%), or unknown extent of disease (20%) at the time of notification of their diagnosis of nonsquamous NSCLC to the New Zealand Cancer Registry. Compared to those with localized or regional disease extent, study patients with distant disease at diagnosis had 40% and 80% increased risks of treatment discontinuation and death, respectively, after commencing treatment with erlotinib or gefitinib. This finding was consistent with previous studies demonstrating the negative impacts of distant metastasis on outcomes from treatment with erlotinib and gefitinib in similar patient groups [44].

Ethnicity was an independent predictor of overall survival, but not of time-to-treatment discontinuation, in this study. Study patients were categorized as Asian (25%), Māori (10%), New Zealand European (52%), Pacific (12%), or unknown or other ethnicity (1%). Time-to-treatment discontinuation was unchanged among these different ethnic groups when compared to New Zealand European group. However, the risk of death was reduced by 20% and 30%, respectively, in the Pacific and Asian groups but unchanged in the other groups compared to New Zealand European. Overall survival may have been impacted by ethnicity in this study independently of the effectiveness of treatment with erlotinib or gefitinib. Ethnicity may have impacted overall survival indirectly through correlated factors, such as smoking status, that vary between ethnic groups and influence the risk of death [50].

### Strengths and Limitations

The strengths of this study include its large population-based sample, internal validity, national generalizability, and unique patient cohort. Only 4 similar analyses [25-28] have included all patients treated in an entire country as far as we are aware. Limitations of the study include those inherent in retrospective study designs or in the use of routinely collected data. The variables available for analysis were limited to those collected routinely during pharmaceutical dispensing and cancer and mortality registration. Some important variables were unavailable or incomplete, such as smoking status, performance status, and clinical stage of disease at the time of commencing treatment with erlotinib and gefitinib, and therefore could not be included in the multivariable analysis. Socioeconomic deprivation was determined by residential area rather than at an individual level, which may have introduced bias. The study did not evaluate the impact of treatments other than erlotinib and gefitinib, which may have influenced overall survival. Safety outcomes were not included in this analysis but will be the subject of subsequent reports.

### Conclusions

Outcomes from treatment with erlotinib and gefitinib in this New Zealand cohort of patients with advanced *EGFR*-mutant nonsquamous NSCLC were comparable to those reported in randomized controlled trials and other large-scale health care system–wide retrospective cohort studies. This nationwide study thereby demonstrated that the therapeutic benefits expected from erlotinib and gefitinib had been achieved in the setting of routine care in New Zealand. In that setting, socioeconomic status, *EGFR* mutation subtype, and disease extent at cancer diagnosis were independent predictors of treatment outcomes.

## Supplementary material

10.2196/65118 Multimedia Appendix 1 Supplementary materials.

## Abbreviations

*EGFR*: epidermal growth factor receptor
HR: hazard ratio
NSCLC: non–small cell lung cancer
PHARMs: Pharmaceutical Information Database
RECORD: Reporting of Studies Conducted Using Observational Routinely-Collected Health Data
