# Supplementary material for: Treatment Outcomes From Erlotinib and Gefitinib in Advanced Epidermal Growth Factor Receptor–Mutated Nonsquamous Non–Small Cell Lung Cancer in Aotearoa New Zealand From 2010 to 2020: Nationwide Whole-of-Patient-Population Retrospective Cohort Study
Source: JMIR Cancer. 2025 Mar 3;11:e65118. doi: 10.2196/65118 (PMC11892703; doi:10.2196/65118)
Supplement: Multimedia Appendix 1 [file cancer-v11-e65118-s001.pdf]

Table S1. Progression-free survival (PFS) for erlotinib and gefitinib treatment arms from randomized clinical trials compared to time-to-treatment discontinuation (TTD) from the current study.

Trial Name	Year	Reference	Treatment	n	Median PFS (% CI) (months)
NEJ002	2010	[1]	Gefitinib	114	10.4 (NS)
WJTOG3405	2010	[2]	Gefitinib	88	9.2 (8.0-13.9)
OPTIMAL	2011	[3]	Erlotinib	82	13.1 (10.57-16.53)
EURTAC	2012	[4]	Erlotinib	86	9.7 (8.4-12.2)
JO25567	2014	[5]	Erlotinib	77	9.7 (5.7-11.1)
ENSURE	2015	[6]	Erlotinib	110	11.0 (NS)
LUX-Lung 7	2016	[7]	Gefitinib	159	10.9 (10.1-12.9)
ARCHER 1050	2017	[8]	Gefitinib	225	9.2 (9.1-11.0)
FLAURA	2018	[9]	Gefitinib/ Erlotinib	183/34	10.2 (15.2-21.4)
RELAY	2019	[10]	Erlotinib	225	12.4 (11.0-13.5)
NEJ026	2019	[11]	Erlotinib	114	13.3 (11.1-15.3)
NEJ009	2019	[12]	Gefitinib	172	11.17 (8.97-13.4)
Noronha	2019	[13]	Gefitinib	176	8.0 (7.0-9.0)
AENEAS	2022	[14]	Gefitinib	215	9.9 (8.3-12.6)
LASER301	2023	[15]	Gefitinib	197	9.7 (9.2-11.3)
			**Total**	**2,257**	
			**Range**		**8.0-13.3**
					**Median TTD (months)**
Current study				752	11.6

References for Table S1.

1. Maemondo M, Inoue A, Kobayashi K, Sugawara S, Oizumi S, Isobe H, et al. Gefitinib or Chemotherapy for Non-Small-Cell Lung Cancer with Mutated EGFR. N Engl J Med. 2010;362(25):2380-8. doi: 10.1056/NEJMoa0909530. PubMed PMID: WOS:000279043800007.

2. Mitsudomi T, Morita S, Yatabe Y, Negoro S, Okamoto I, Tsurutani J, et al. Gefitinib versus cisplatin plus docetaxel in patients with non-small-cell lung cancer harbouring mutations of the epidermal growth factor receptor (WJTOG3405): an open label, randomised phase 3 trial. Lancet Oncology. 2010;11(2):121-8. doi: 10.1016/s1470-2045(09)70364-x. PubMed PMID: WOS:000275152900019.

3. Zhou CC, Wu YL, Chen GY, Feng JF, Liu XQ, Wang CL, et al. Erlotinib versus chemotherapy as first-line treatment for patients with advanced EGFR mutation-positive non-small-cell lung cancer (OPTIMAL, CTONG-0802): a multicentre, open-label, randomised, phase 3 study. Lancet Oncology. 2011;12(8):735-42. doi: 10.1016/s1470-2045(11)70184-x. PubMed PMID: WOS:000293272100024.

4. Rosell R, Carcereny E, Gervais R, Vergnenegre A, Massuti B, Felip E, et al. Erlotinib versus standard chemotherapy as first-line treatment for European patients with advanced EGFR mutation-positive non-small-cell lung cancer (EURTAC): a multicentre, open-label, randomised phase 3 trial. Lancet Oncology. 2012;13(3):239-46. doi: 10.1016/s1470-2045(11)70393-x. PubMed PMID: WOS:000300880600031.

5. Seto T, Kato T, Nishio M, Goto K, Atagi S, Hosomi Y, et al. Erlotinib alone or with bevacizumab as first-line therapy in patients with advanced non-squamous non-small-cell lung cancer harbouring *EGFR* mutations (JO25567): an open-label, randomised, multicentre, phase 2 study. Lancet Oncology. 2014;15(11):1236-44. doi: 10.1016/s1470-2045(14)70381-x. PubMed PMID: WOS:000343096800049.

6. Wu Y-L, Zhou C, Liam C-K, Wu G, Liu X, Zhong Z, et al. First-line erlotinib versus gemcitabine/cisplatin in patients with advanced EGFR mutation-positive non-small-cell lung cancer: analyses from the phase III, randomized, open-label, ENSURE study. Annals of Oncology. 2015;26(9):1883-9. doi: 10.1093/annonc/mdv270.

7. Park K, Tan EH, O'Byrne K, Zhang L, Boyer M, Mok T, et al. Afatinib versus gefitinib as first-line treatment of patients with EGFR mutation-positive non-small-cell lung cancer (LUX-Lung 7): a phase 2B, open-label, randomised controlled trial. Lancet Oncology. 2016;17(5):577-89. doi: 10.1016/s1470-2045(16)30033-x. PubMed PMID: WOS:000374829800049.

8. Wu Y-L, Cheng Y, Zhou X, Lee KH, Nakagawa K, Niho S, et al. Dacomitinib versus gefitinib as first-line treatment for patients with EGFR-mutation-positive non-small-cell lung cancer (ARCHER 1050): a randomised, open-label, phase 3 trial. Lancet Oncology. 2017;18(11):1454-66. doi: 10.1016/s1470-2045(17)30608-3. PubMed PMID: WOS:000414134100053.

9. Soria JC, Ohe Y, Vansteenkiste J, Reungwetwattana T, Chewaskulyong B, Lee KH, et al. Osimertinib in Untreated EGFR-Mutated Advanced Non-Small-Cell Lung Cancer. N Engl J Med. 2018;378(2):113-25. doi: 10.1056/NEJMoa1713137. PubMed PMID: WOS:000419971600005.

10. Nakagawa K, Garon EB, Seto T, Nishio M, Aix SP, Paz-Ares L, et al. Ramucirumab plus erlotinib in patients with untreated, EGFR-mutated, advanced non-small-cell lung cancer (RELAY): a randomised, double-blind, placebo-controlled, phase 3 trial. Lancet Oncology. 2019;20(12):1655-69. doi: 10.1016/S1470-2045(19)30634-5. PubMed PMID: WOS:000500750400046.

11. Saito H, Fukuhara T, Furuya N, Watanabe K, Sugawara S, Iwasawa S, et al. Erlotinib plus bevacizumab versus erlotinib alone in patients with *EGFR*-positive advanced non-squamous non-small-cell lung cancer (NEJ026): interim analysis of an open-label, randomised, multicentre, phase 3 trial. Lancet Oncology. 2019;20(5):625-35. doi: 10.1016/s1470-2045(19)30035-x. PubMed PMID: WOS:000466380000056.

12. Hosomi Y, Morita S, Sugawara S, Kato T, Fukuhara T, Gemma A, et al. Gefitinib Alone Versus Gefitinib Plus Chemotherapy for Non-Small-Cell Lung Cancer With Mutated Epidermal Growth Factor Receptor: NEJ009 Study. Journal of Clinical Oncology. 2020;38(2):115-+. doi: 10.1200/jco.19.01488. PubMed PMID: WOS:000508198400003.

13. Noronha V, Patil VM, Joshi A, Menon N, Chougule A, Mahajan A, et al. Gefitinib Versus Gefitinib Plus Pemetrexed and Carboplatin Chemotherapy in *EGFR*-Mutated Lung Cancer. Journal of Clinical Oncology. 2020;38(2):124-+. doi: 10.1200/jco.19.01154. PubMed PMID: WOS:000508198400004.

14. Lu S, Dong X, Jian H, Chen J, Chen G, Sun Y, et al. AENEAS: A Randomized Phase III Trial of Aumolertinib Versus Gefitinib as First-Line Therapy for Locally Advanced or MetastaticNon–Small-Cell Lung Cancer With EGFR Exon 19 Deletion or L858R Mutations. Journal of Clinical Oncology. 2022;40(27):3162-71. doi: 10.1200/JCO.21.02641.

15. Cho BC, Ahn MJ, Kang JH, Soo RA, Reungwetwattana T, Yang JCH, et al. Lazertinib Versus Gefitinib as First-Line Treatment in Patients With *EGFR*-Mutated Advanced Non-Small-Cell Lung Cancer: Results From LASER301. Journal of Clinical Oncology. 2023;41(26):4208-+. doi: 10.1200/jco.23.00515. PubMed PMID: WOS:001069556400005.

Table S2. Nationwide or healthcare system-wide observational studies reporting progression-free survival, or proxies of progression-free survival, from the time of commencing treatment with erlotinib or gefitinib in patients in advanced EGFR-mutant lung cancer.

First Author	Li	Hsieh		Pluzanski		Chen		Manninen
Date	2019	2020		2020		2021		2023
Reference	[1]	[2]		[3]		[4]		[5]
Study Design	Population-based retrospective cohort analysis	Population-based retrospective cohort analysis		Population-based retrospective cohort analysis		Population-based retrospective cohort analysis		Population-based retrospective cohort analysis
Country	USA	Taiwan		Poland		Taiwan		Finland
Setting	Patients treated at USA cancer centres contributing to the Flatiron Health Cloud-based Electronic Health Record-derived database	National healthcare system		National healthcare system		National healthcare system		National healthcare system
Population, eligibility, period	EGFR-mutant NSCLC stage IIIB/IV exon19 or L858R 2011-2016	Patients with EGFR-mutant NSCLC stage IIIB or IV; PS 0-2; treated with EGFR-TKI between 2011 and 2015		Treatment-naïve EGFR-mutant stage IIB/IV NSCLC started EGFR-TKI 2012-2016		EGFR-mutant advanced lung adenocarcinoma treatment with EGFR-TKI 2013-2017		Patients with activating EGFR mutations dispensed EGFR-TKI with lung cancer registry data 2011-2020
Data Sources	Flatiron Health cloud-based electronic health record database used by 265 US cancer clinics	National administrative datasets (Taiwan Cancer Registry; Taiwan National Health Insurance database; National Death Registry)		National Health Fund TP Database		Taiwan Health Insurance Research Database		National Prescription, Cancer Registry and Death Statistic databases
Sampling methodology	Whole-of-patient-population sample	Whole-of-patient-population sample		Whole-of-patient-population sample		Whole-of-patient-population sample		Whole-of-patient-population sample
EGFR-TKI	Erlotinib	Gefitinib	Erlotinib	Gefitinib	Erlotinib	Gefitinib	Erlotinib	Gefitinib
n	593	3982	1207	253	255	3695	3301	238
Age (mean)	69	66.1	64.9	67	68	70.4	66.8	NR
Female (%)	69.1	66	56	70	63	70.4	58.6	NR
PFS or proxy	Time to next treatment	Time to treatment failure		PFS		Time to treatment failure		Time on treatment
Median (95% CI)	13.1 (12.1-14.3)	11.9 (11.5-12.3)	12.7 (12- 13.1)	10.3 (8.4-12.0)	12.1 (9.9-14.8)	9.7 (NR)	9.7 (NR)	11.9 (NR)

Overall survival, median (95% CI)	23.2 (21.2-24.9)	22.8 (21.9-23.5)	23.9 (22.6- 25.8)	17.5 (15.2-20.3)	20.4 (17.5-27.8)	20.9 (NR)	21.4 (NR)	NR
EGFR mutation subtypes (%)	Exon 19 Del (55.0%); L858R (45.0%)	NR		NR		NR		NR
Correlates	NR	Age, sex, stage, histology, performance status, smoking		NR		Age, sex, comorbidity, brain metastasis		Sex

References for Table S2

1. Li YL, Appius A, Pattipaka T, Feyereislova A, Cassidy A, Ganti AK. Real-world management of patients with epidermal growth factor receptor (*EGFR*) mutation-positive non-small-cell lung cancer in the USA. Plos One. 2019;14(1). doi: 10.1371/journal.pone.0209709. PubMed PMID: WOS:000454952800023.

2. Hsieh Y-Y, Fang W-T, Lo Y-W, Chen Y-H, Chien L-N. Comparing the effectiveness of different EGFR-TKIs in patients with EGFR mutant non–small-cell lung cancer: A retrospective cohort study in Taiwan. Int J Cancer. 2020;147(4):1107-16. doi: https://doi.org/10.1002/ijc.32841.

3. Pluzanski A, Krzakowski M, Kowalski D, Dziadziuszko R. Real-world clinical outcomes of first-generation and second-generation epidermal growth factor receptor tyrosine kinase inhibitors in a large cohort of European non-small-cell lung cancer patients. Esmo Open. 2020;5(6). doi: 10.1136/esmoopen-2020-001011. PubMed PMID: WOS:000591346900002.

4. Chen P-Y, Wang C-C, Hsu C-N, Chen C-Y. Association of EGFR Tyrosine Kinase Inhibitor Treatment With Progression-Free Survival Among Taiwanese Patients With Advanced Lung Adenocarcinoma and EGFR Mutation. Frontiers in Pharmacology. 2021;12.

5. Manninen O, Puuniemi L, Iivanainen S, Arffman M, Kaarteenaho R, Koivunen JP. Treatment outcomes of non-small cell lung cancers treated with EGFR tyrosine kinase inhibitors: a real-world cohort study. Acta Oncologica. 2023;62(12):1854-61. doi: 10.1080/0284186X.2023.2274481.

Table S3. Sensitivity analyses of less stringent criteria for factor inclusion in multivariable analyses of time-to-treatment discontinuation of P<0.12 (first table) or any P value (second table).

		Univariable analysis			Multivariable analysis		
		HR	(95% CI)	P value	HR	(95% CI)	P value
EGFR-TKI	Gefitinib	1.1	(1.0-1.3)	0.074	1.1	(1.0-1.3)	0.096
	Erlotinib	1.0			1.0		
Age	<65 years	1.0	(0.9-1.2)	0.633			
	65+ years	1.0					
Sex	Male	1.0	(0.8-1.1)	0.744			
	Female	1.0					
Ethnicity	NZ European	1.0					
	Māori	1.1	(0.9-1.4)	0.371			
	Pacific	1.0	(0.8-1.2)	0.760			
	Asian	0.9	(0.7-1.0)	0.135			
	Other & Unknown	0.8	(0.4-1.7)	0.581			
Region	Northern	1.0					
	Midland	1.1	(0.9-1.4)	0.446			
	Others	1.0	(0.9-1.2)	0.950			
Diagnosis year	2010-2013	1.0	(0.8-1.2)	0.920			
	2014-2016	0.9	(0.8-1.1)	0.174			
	2017-2020	1.0					
Morphology	Adenocarcinoma	1.0			1.0		
	Unspecified and other	0.8	(0.6-1.0)	0.039	0.9	(0.7-1.1)	0.308
Basis of diagnosis	Histology	1.0					
	Cytology	1.1	(0.9-1.3)	0.292			
	Other	0.9	(0.5-1.4)	0.579			
Extent	Localised/regional	1.0			1.0		
	Distant	1.5	(1.2-1.8)	<0.001	1.4	(1.1-1.7)	0.001

	Unknown	0.9	(0.7-1.1)	0.384	0.9	(0.7-1.1)	0.364
Deprivation	NZDep 1-4	1.0			1.0		
	NZDep 5-7	1.2	(1.0-1.5)	0.016	1.2	(1.0-1.5)	0.038
	NZDep 8-10	1.3	(1.1-1.6)	0.004	1.3	(1.1-1.6)	0.002
Rurality	Urban	1.0					
	Rural	1.1	(0.9-1.4)	0.387			
Comorbidity	No	1.2	(1.0-1.4)	0.111	1.2	(1.0-1.4)	0.117
	Yes	1.0			1.0		
EGFR type	19 del	1.0			1.0		
	21 l858r	1.3	(1.1-1.5)	0.001	1.3	(1.1-1.6)	<0.001
	uncommon/actionable	1.2	(0.9-1.6)	0.166	1.3	(1.0-1.7)	0.051

		Univariable analysis			Multivariable analysis		
		HR	(95% CI)	P value	HR	(95% CI)	P value
EGFR-TKI	Gefitinib	1.1	(1.0-1.3)	0.074	1.2	(1.0-1.4)	0.062
	Erlotinib	1.0			1.0		
Age	<65 years	1.0	(0.9-1.2)	0.633	1.0	(0.9-1.2)	0.710
	65+ years	1.0			1.0		
Sex	Male	1.0	(0.8-1.1)	0.744	1.0	(0.8-1.1)	0.681
	Female	1.0			1.0		
Ethnicity	NZ European	1.0			1.0		
	Māori	1.1	(0.9-1.4)	0.371	1.2	(0.9-1.5)	0.258
	Pacific	1.0	(0.8-1.2)	0.760	0.8	(0.6-1.1)	0.130
	Asian	0.9	(0.7-1.0)	0.135	0.9	(0.7-1.1)	0.180
	Other & Unknown	0.8	(0.4-1.7)	0.581	0.8	(0.4-1.8)	0.643
Region	Northern	1.0			1.0		
	Midland	1.1	(0.9-1.4)	0.446	1.0	(0.8-1.3)	0.867

	Others	1.0	(0.9-1.2)	0.950	0.9	(0.8-1.1)	0.253
Diagnosis year	2010-2013	1.0	(0.8-1.2)	0.920	0.9	(0.7-1.2)	0.515
	2014-2016	0.9	(0.8-1.1)	0.174	0.9	(0.8-1.0)	0.145
	2017-2020	1.0			1.0		
Morphology	Adenocarcinoma	1.0			1.0		
	Unspecified and other	0.8	(0.6-1.0)	0.039	0.9	(0.7-1.1)	0.283
Basis of diagnosis	Histology	1.0			1.0		
	Cytology	1.1	(0.9-1.3)	0.292	1.0	(0.8-1.1)	0.676
	Other	0.9	(0.5-1.4)	0.579	0.8	(0.5-1.3)	0.397
Extent	Localised/regional	1.0			1.0		
	Distant	1.5	(1.2-1.8)	<0.001	1.4	(1.2-1.8)	0.001
	Unknown	0.9	(0.7-1.1)	0.384	0.9	(0.7-1.2)	0.433
Deprivation	NZDep 1-4	1.0			1.0		
	NZDep 5-7	1.2	(1.0-1.5)	0.016	1.3	(1.0-1.5)	0.020
	NZDep 8-10	1.3	(1.1-1.6)	0.004	1.3	(1.1-1.6)	0.004
Rurality	Urban	1.0			1.0		
	Rural	1.1	(0.9-1.4)	0.387	1.1	(0.9-1.4)	0.520
Comorbidity	No	1.2	(1.0-1.4)	0.111	1.2	(1.0-1.4)	0.105
	Yes	1.0			1.0		
EGFR type	19 del	1.0			1.0		
	21 L858R	1.3	(1.1-1.5)	0.001	1.4	(1.2-1.6)	<0.001
	uncommon/actionable	1.2	(0.9-1.6)	0.166	1.3	(1.0-1.6)	0.105

Table S4. Sensitivity analyses of less stringent criteria for factor inclusion in multivariable analyses of overall survival of P<0.12 (first table) or any P value (second table).

		Univariable analysis			Multivariable analysis		
		HR	(95% CI)	P value	HR	(95% CI)	P value
EGFR-TKI	Gefitinib	1.2	(1.0-1.4)	0.020	1.2	(1.0-1.4)	0.095
	Erlotinib	1.0			1.0		
Age	<65 years	0.9	(0.7-1.0)	0.048	0.9	(0.7-1.0)	0.127
	65+ years	1.0			1.0		
Sex	Male	1.1	(0.9-1.3)	0.482			
	Female	1.0					
Ethnicity	NZ European	1.0			1.0		
	Māori	1.2	(0.9-1.5)	0.263	1.3	(1.0-1.7)	0.052
	Pacific	0.8	(0.6-1.0)	0.059	0.8	(0.6-1.0)	0.055
	Asian	0.7	(0.5-0.8)	<0.001	0.7	(0.6-0.9)	<0.001
	Other & Unknown	0.5	(0.2-1.3)	0.148	0.5	(0.2-1.3)	0.169
Region	Northern	1.0			1.0		
	Midland	1.2	(0.9-1.5)	0.182	1.1	(0.8-1.4)	0.630
	Others	1.4	(1.1-1.6)	<0.001	1.2	(1.0-1.5)	0.060
Diagnosis year	2010-2013	1.2	(0.9-1.4)	0.236			
	2014-2016	1.0	(0.8-1.2)	0.819			
	2017-2020	1.0					
Morphology	Adenocarcinoma	1.0			1.0		
	Unspecified and other	0.7	(0.6-1.0)	0.018	0.9	(0.7-1.2)	0.408
Basis of diagnosis	Histology	1.0					
	Cytology	1.1	(0.9-1.3)	0.411			
	Other	1.4	(0.9-2.3)	0.162			
Extent	Localised/regional	1.0			1.0		
	Distant	1.7	(1.4-2.2)	<0.001	1.8	(1.4-2.2)	<0.001

	Unknown	1.0	(0.8-1.3)	0.978	1.0	(0.7-1.3)	0.824
Deprivation	NZDep 1-4	1.0			1.0		
	NZDep 5-7	1.4	(1.2-1.7)	0.001	1.3	(1.1-1.6)	0.005
	NZDep 8-10	1.4	(1.1-1.7)	0.001	1.4	(1.1-1.7)	0.004
Rurality	Urban	1.0			1.0		
	Rural	1.2	(1.0-1.5)	0.109	1.1	(0.8-1.4)	0.589
Comorbidity	No	1.0	(0.9-1.3)	0.666			
	Yes	1.0					
EGFR type	19 del	1.0			1.0		
	21 L858R	1.4	(1.2-1.6)	<0.001	1.5	(1.2-1.7)	<0.001
	uncommon/actionable	1.3	(1.0-1.7)	0.086	1.2	(0.9-1.6)	0.179

		Univariable analysis			Multivariable analysis		
		HR	(95% CI)	P value	HR	(95% CI)	P value
EGFR-TKI	Gefitinib	1.2	(1.0-1.4)	0.020	1.1	(0.9-1.4)	0.273
	Erlotinib	1.0			1.0		
Age	<65 years	0.9	(0.7-1.0)	0.048	0.8	(0.7-1.0)	0.064
	65+ years	1.0			1.0		
Sex	Male	1.1	(0.9-1.3)	0.482	1.1	(0.9-1.3)	0.334
	Female	1.0					
Ethnicity	NZ European	1.0			1.0		
	Māori	1.2	(0.9-1.5)	0.263	1.3	(1.0-1.7)	0.064
	Pacific	0.8	(0.6-1.0)	0.059	0.8	(0.6-1.01)	0.104
	Asian	0.7	(0.5-0.8)	<0.001	0.7	(0.6-0.9)	0.001
	Other & Unknown	0.5	(0.2-1.3)	0.148	0.5	(0.2-1.3)	0.157
Region	Northern	1.0			1.0		
	Midland	1.2	(0.9-1.5)	0.182	1.1	(0.8-1.5)	0.474

	Others	1.4	(1.1-1.6)	<0.001	1.2	(1.0-1.5)	0.039
Diagnosis year	2010-2013	1.2	(0.9-1.4)	0.236	1.2	(0.9-1.5)	0.291
	2014-2016	1.0	(0.8-1.2)	0.819	1.0	(0.8-1.2)	0.931
	2017-2020	1.0			1.0		
Morphology	Adenocarcinoma	1.0			1.0		
	Unspecified and other	0.7	(0.6-1.0)	0.018	0.9	(0.7-1.1)	0.317
Basis of diagnosis	Histology	1.0			1.0		
	Cytology	1.1	(0.9-1.3)	0.411	1.0	(0.8-1.1)	0.636
	Other	1.4	(0.9-2.3)	0.162	1.3	(0.8-2.1)	0.391
Extent	Localised/regional	1.0			1.0		
	Distant	1.7	(1.4-2.2)	<0.001	1.7	(1.4-2.2)	<0.001
	Unknown	1.0	(0.8-1.3)	0.978	1.0	(0.7-1.3)	0.804
Deprivation	NZDep 1-4	1.0			1.0		
	NZDep 5-7	1.4	(1.2-1.7)	0.001	1.4	(1.1-1.7)	0.004
	NZDep 8-10	1.4	(1.1-1.7)	0.001	1.4	(1.1-1.7)	0.002
Rurality	Urban	1.0			1.0		
	Rural	1.2	(1.0-1.5)	0.109	1.1	(0.8-1.4)	0.651
Comorbidity	No	1.0	(0.9-1.3)	0.666	1.1	(0.9-1.4)	0.243
	Yes	1.0					
EGFR type	19 del	1.0			1.0		
	21 L858R	1.4	(1.2-1.6)	<0.001	1.5	(1.2-1.8)	<0.001
	uncommon/actionable	1.3	(1.0-1.7)	0.086	1.2	(0.9-1.6)	0.205

Table S5. The REporting of studies Conducted using Observational Routinely-collected health Data (RECORD) Statement – checklist of items, extended from the STROBE statement, that should be reported in observational studies using routinely collected health data.

	Item No.	STROBE items	Location in manuscript where items are reported	RECORD items	Location in manuscript where items are reported
**Title and abstract**					
	1	(a) Indicate the study's design with a commonly used term in the title or the abstract (b) Provide in the abstract an informative and balanced summary of what was done and what was found	1, 4	RECORD 1.1: The type of data used should be specified in the title or abstract. When possible, the name of the databases used should be included.<br><br>RECORD 1.2: If applicable, the geographic region and timeframe within which the study took place should be reported in the title or abstract.	1, 4<br><br>4

				RECORD 1.3: If linkage between databases was conducted for the study, this should be clearly stated in the title or abstract.	4
**Introduction**					
Background rationale	2	Explain the scientific background and rationale for the investigation being reported	6, 7		
Objectives	3	State specific objectives, including any prespecified hypotheses	7		
**Methods**					
Study Design	4	Present key elements of study design early in the paper	8		
Setting	5	Describe the setting, locations, and relevant dates, including periods of recruitment,	8		

		exposure, follow-up, and data collection			
Participants	6	*(a) Cohort study* - Give the eligibility criteria, and the sources and methods of selection of participants. Describe methods of follow-up<br><br>*Case-control study* - Give the eligibility criteria, and the sources and methods of case ascertainment and control selection. Give the rationale for the choice of cases and controls<br><br>*Cross-sectional study* - Give the eligibility criteria, and the sources and methods of selection of participants<br><br>*(b) Cohort study* - For matched studies, give matching criteria and number of exposed and unexposed	8, 14	RECORD 6.1: The methods of study population selection (such as codes or algorithms used to identify subjects) should be listed in detail. If this is not possible, an explanation should be provided.<br><br>RECORD 6.2: Any validation studies of the codes or algorithms used to select the population should be referenced. If validation was conducted for this study and not published elsewhere, detailed methods and results should be provided.<br><br>RECORD 6.3: If the study involved linkage of databases, consider use of a flow diagram or other graphical display to demonstrate the data linkage process, including the number of	8, 14<br><br>11<br><br>14

		Case-control study - For matched studies, give matching criteria and the number of controls per case		individuals with linked data at each stage.	
Variables	7	Clearly define all outcomes, exposures, predictors, potential confounders, and effect modifiers. Give diagnostic criteria, if applicable.	9-11	RECORD 7.1: A complete list of codes and algorithms used to classify exposures, outcomes, confounders, and effect modifiers should be provided. If these cannot be reported, an explanation should be provided.	9-11
Data sources/ measurement	8	For each variable of interest, give sources of data and details of methods of assessment (measurement).<br><br>Describe comparability of assessment methods if there is more than one group	10		
Bias	9	Describe any efforts to address potential sources of bias	11		
Study size	10	Explain how the study size was arrived at	14		

Quantitative variables	11	Explain how quantitative variables were handled in the analyses. If applicable, describe which groupings were chosen, and why	10, 11		
Statistical methods	12	(a) Describe all statistical methods, including those used to control for confounding<br><br>(b) Describe any methods used to examine subgroups and interactions<br><br>(c) Explain how missing data were addressed<br><br>(d) *Cohort study* - If applicable, explain how loss to follow-up was addressed<br><br>*Case-control study* - If applicable, explain how matching of cases and controls was addressed<br><br>*Cross-sectional study* - If applicable, describe analytical	11, 12<br><br>11, 12<br><br>12<br><br>NA		

		methods taking account of sampling strategy<br><br>(e) Describe any sensitivity analyses			
Data access and cleaning methods		..		RECORD 12.1: Authors should describe the extent to which the investigators had access to the database population used to create the study population.<br><br>RECORD 12.2: Authors should provide information on the data cleaning methods used in the study.	8<br><br>10, 11
Linkage		..		RECORD 12.3: State whether the study included person-level, institutional-level, or other data linkage across two or more databases. The methods of linkage and methods of linkage quality evaluation should be provided.	9
**Results**					

Participants	13	(a) Report the numbers of individuals at each stage of the study (*e.g.*, numbers potentially eligible, examined for eligibility, confirmed eligible, included in the study, completing follow-up, and analysed)<br><br>(b) Give reasons for non-participation at each stage.<br><br>(c) Consider use of a flow diagram	12-14	RECORD 13.1: Describe in detail the selection of the persons included in the study (*i.e.,* study population selection) including filtering based on data quality, data availability and linkage. The selection of included persons can be described in the text and/or by means of the study flow diagram.	12-14
Descriptive data	14	(a) Give characteristics of study participants (*e.g.*, demographic, clinical, social) and information on exposures and potential confounders<br><br>(b) Indicate the number of participants with missing data for each variable of interest<br><br>(c) *Cohort study* - summarise follow-up time (*e.g.*, average and total amount)	15-16<br><br>15<br><br>17, 19		

Outcome data	15	*Cohort study* - Report numbers of outcome events or summary measures over time<br><br>*Case-control study* - Report numbers in each exposure category, or summary measures of exposure<br><br>*Cross-sectional study* - Report numbers of outcome events or summary measures	17-21		
Main results	16	(a) Give unadjusted estimates and, if applicable, confounder-adjusted estimates and their precision (e.g., 95% confidence interval). Make clear which confounders were adjusted for and why they were included<br><br>(b) Report category boundaries when continuous variables were categorized<br><br>(c) If relevant, consider translating estimates of relative	17-20<br><br>15, 16		

		risk into absolute risk for a meaningful time period			
Other analyses	17	Report other analyses done—e.g., analyses of subgroups and interactions, and sensitivity analyses	17, 19		
**Discussion**					
Key results	18	Summarise key results with reference to study objectives	22-24		
Limitations	19	Discuss limitations of the study, taking into account sources of potential bias or imprecision. Discuss both direction and magnitude of any potential bias	24, 25	RECORD 19.1: Discuss the implications of using data that were not created or collected to answer the specific research question(s). Include discussion of misclassification bias, unmeasured confounding, missing data, and changing eligibility over time, as they pertain to the study being reported.	24, 25
Interpretation	20	Give a cautious overall interpretation of results considering objectives,	25		

		limitations, multiplicity of analyses, results from similar studies, and other relevant evidence			
Generalisability	21	Discuss the generalisability (external validity) of the study results	24, 25		
**Other Information**					
Funding	22	Give the source of funding and the role of the funders for the present study and, if applicable, for the original study on which the present article is based	26		
Accessibility of protocol, raw data, and programming code		..		RECORD 22.1: Authors should provide information on how to access any supplemental information such as the study protocol, raw data, or programming code.	25

*Reference: Benchimol EI, Smeeth L, Guttmann A, Harron K, Moher D, Petersen I, Sørensen HT, von Elm E, Langan SM, the RECORD Working Committee. The REporting of studies Conducted using Observational Routinely-collected health Data (RECORD) Statement. *PLoS Medicine* 2015; in press.

*Checklist is protected under Creative Commons Attribution (CC BY) license.
